# Conformational Dynamics in Insulin Receptor Kinase Reveals a Type III Allosteric Pocket

**DOI:** 10.1101/2025.05.10.653287

**Published:** 2025-05-15

**Authors:** Jyoti Verma, Harish Vashisth

## Abstract

Allosteric modulation of kinases is a promising approach for pharmacological intervention, particularly for designing selective kinase modulators. However, the structural information on allosteric pockets remains limited for most kinases in the human kinome. In this work, we present comprehensive characterization of a type III allosteric pocket in the insulin receptor kinase (IRK) by uncovering its structural features and conformational dynamics. Specifically, we used microsecond-scale atomistic molecular dynamics (MD) simulations to investigate apo and inhibitor-bound IRK structures. Our findings suggest that the type III allosteric site is a “back pocket” in IRK, sandwiched between the N-terminal and the C-terminal lobes, beneath the *α* C-helix and the *β* - sheets of the N-terminal lobe. It has a hydrophobic cleft composed of both aliphatic and aromatic non-polar residues and a charge center to facilitate electrostatic interactions. We explored the binding of an experimentally known IRK inhibitor to the newly discovered allosteric pocket. Our results indicate that the *α* C-helix adopts an “out” conformation stabilized by the inhibitor which promotes an inactive conformation of the kinase. Furthermore, we observed a helical intermediate formation in the activation loop and a stable “DFG-out” conformation in both inhibitor-bound and unbound states. Our results also suggest that the residue M1051 in IRK functions as a gatekeeper residue, essential for maintaining the structural integrity of the *α* C-helix and regulating the binding of the allosteric inhibitor. Our findings are relevant for developing allosteric IRK modulators and informing therapeutic strategies targeting proteins in the insulin receptor family.

## Full Text Availability

The license terms selected by the author(s) for this preprint version do not permit archiving in PMC. The full text is available from the preprint server.

